# Illinois State Dental Society

**Published:** 1884-09

**Authors:** 


### ILLINOIS STATE DENTAL SOCIETY.

### TWENTIETH ANNUAL MEETING.

REPORTED EXPRESSLY FOR THE INDEPENDENT PRACTITIONER.

(Continued from page 444.)

### THE ORIGIN OF DEFECTIVE TOOTH STRUCTURE, KNOWN AS PITTED, FURROWED OR CRIBRIFORM ENAMEL.

Dr. W. H. Eames read a very able and instructive paper upon this subject. He considered under two general heads the various forms of defective enamel hitherto classed as rocky, ridged, furrowed, cribriform, pitted and grooved. These formations were generally attributed to one cause—arrested development. He insisted that not to constitutional disturbances, such as measles, eruptive fevers, eclampsia or syphilis, is the defective enamel structure due, but to a defect in the formative organ.

He next called attention to the fissures or grooves in the coronal surface of molars and bicuspids, and said that they are also attributed to arrested development. He affirmed that, as Dr. Black of this Society has clearly shown, the defect is manifestly not due to a failure to coalesce, but rather results from rupture of the enamel organ at this point.

Of the second class of cases of defective enamel, continued the speaker, the "non-congenital or accidental," as was suggested in my paper of last year I believe a large number to be due to the abnormal action of the so-called absorption organ. Is it not possible that, having performed their work of removing the roots of the temporary teeth and the alveoli, they continue to act and remove the enamel cuticle and dissolve out the lime from the freshly amelified enamel rods with which they come into immediate contact at the gingival border?

Upon the conclusion of Dr. Eames' paper, the reading of which was attentively listened to throughout, the society took a recess until two o'clock P. M.

AFTERNOON SESSION.

The regular order, discussion of Dr. Eames' paper, was announced, and Dr. L. C. Ingersoll was invited to open the subject.

*Dr. Ingersoll.*—The subject presented in the very lucid and able paper by Prof. Eames is too large to be fully covered within the limits of a discussion upon an occasion like this. I can, therefore, only allude to what I consider the more salient and important points.

The imperfect enamel tissue appearing in single spots, pits or wells, in various locations, I am willing to concede is due to congenital causes. The theory of Prof. Eames is ingenious, but cannot, I think, apply to the first molars and bicuspids. But we do agree in this—that the old theory is wrong—and as I proceed, you will see where we disagree.

The strongest advance in the historical argument in support of the old theory of congenital disease is found in the oft-repeated statement, "the child's mother told me so." Yet look at the portentous array of diseases to which children are subject in early life, and you will see that it is almost impossible for the child not to have had some of these diseases before attaining the age of five years. So when the mother is asked "Did not the child have such and such a disease at a certain age?" she immediately and truthfully answers "Yes." The child may have had measles, whooping-cough, eclampsia, scarlatina, eruptive fever, gastric fever, scald head, or any of a long list of exanthematous diseases to which children are especially liable at the assumed period of age mentioned by the inquirer. And we are told that these markings on the teeth enable one to determine the age when this constitutional disturbance occurred.

Now what does the average dentist say relative to the formation of the pits and grooves of the teeth? He assumes that when one groove was formed the child had, say scarlatina, at another time whooping-cough, at another chicken, or possibly, small pox; next, perhaps eclampsia, and so on to the required number of diseases corresponding with the number of grooves found in the child's teeth. If anything can be found in the whole range of etiology more ridiculous than this, I should like to have it shown.

If we carefully examine the commonly accepted theory, we shall find that it is based on the hypothesis that, while the enamel is being formed, a line of enamel cells, extending horizontally across the labial face of the tooth, ceases for a time the process of amelification, and the result of this suspension of function is a well marked line or groove, or irregularly formed pits, which come into view when the tooth emerges from the gum. Dr. Eames stated in his paper on the subject, read last year, that enamel is deposited in continuous sheets over the entire cap of dentine.

Now I maintain that the doctrine of alternate vital action is the physiological law of all development. The working out of this law is one of the economies of nature. Observe the development of the buds and the leaves of the tree. The roots have shot out under the ground; then they cease to grow, and the leaves take their turn at development. In the case of the peach, the young fruit grows rapidly until it reaches the size of a robin's egg, when comes a period of suspended growth. During this period of apparent rest the life force of the plant is not suspended, but is engaged in the work of developing some other part. At this time the stone, which before was pulpy, becomes hard and stony, and the force of the tree is expanded in the development of its seed. Alternate development is, therefore, a physiological law, and arrested growth or tissue impairment is not dependent upon the occurrence of constitutional disease.

The most puzzling question involved in the old hypothesis, is how to account for the occurrence of *parallel* grooves, disposed at regular intervals, and sometimes numbering as many as five or six.

The theory that I propose is that these markings do not occur during follicular growth and development, but are the result of action which takes place after the crown of the tooth has penetrated the gum. The groove is formed by chemical erosion of the enamel, and the line of action corresponds with the margin of the gum. This action takes place on the labial and buccal surfaces, and sometimes, when conditions are favorable, on the lingual also. This destructive process may be carried on at any stage of the eruption or emergence of the crown, and will occur at intervals by reason of the operations of the physiological law which governs both plant and animal development. The law of *alternate* vital action prevails in both kingdoms; that is, active development alternates with suspended or diminished development.

An acrid fluid, capable in its nascent condition of dissolving the lime salts of the enamel, is often found at the margins of the gums, which during the stay in the emergence of the tooth forms a groove by erosion. When active emergence is renewed, the moving tooth is not liable to be thus affected; but on the return of the cessation of emergence, erosion again takes place, and a second groove is the result.

The subject being passed, the discussion of Dr. Judd's paper on Inflammation was declared in order.

*Dr. Crouse*—I would like to ask Dr. Judd a question concerning dental caries in relation to inflammation.

*Dr. Judd*—I stated Dr. Stricker's theory of a return to original conditions, but I said I did not agree with it. In fact, I do not think the theory is endorsed by many scientific men.

*Dr. Crouse*—I would like Dr. Taft's opinion; whether he agrees with or endorses the theory.

*Dr. Taft*—The hypothesis is all nonsense. It is impossible to return to that condition. But the expression is used in referring to decaying teeth. I have not been able to get a good idea of the theory, for the reason that the champions of it themselves differ in expression.

*Dr. Crouse*—I will ask Dr. Black about that theory of Stricker. I understand he favors it.

*Dr. Black*—I will answer like the little boy—"There's nothing in it."

THURSDAY MORNING CLINICS.

The time was occupied until noon in clinics, which were held in the ladies' ordinary at the Leland Hotel.

*Dr. J. A. Swasey*, of Chicago—Illustrated the use of Robinson's metal in conjunction with gold foil. He operated on a lower left inferior molar, which contained a large, complicated cavity. The body of the filling was of Robinson's metal, finishing with gold foil, Nos. 4 and 10. The clinic was made for the purpose of showing that the combination of the different materials would form a better filling in teeth with large contours and deep cervical walls, than either material alone.

*Dr. Jas. G. Reed*, of Chicago—Demonstrated the method of filling root-canals with gutta percha dissolved in chloroform.

*Dr. Conrad*, of St. Louis—Mounted a porcelain crown on the root of a cuspid. In some particulars the method he followed was an original one.

*Dr. E. Honsinger*, of Chicago—Filled a posterior approximal cavity in a second inferior molar. This was filled with Watts' crystal gold, and the operation occupied three hours.

*Dr. H. H. Townsend*, of Pontiac—Operated on a left superior first bicuspid, from which the pulp had been removed.

The root canal was filled with white gutta-percha solution. The cavity was compound, being upon the distal and across the grinding surfaces, and was filled with gold. The operation was completed with Williams' rolled gold No. 120.

*Dr. Edgar D. Swain*, of Chicago—Mounted a gold crown on a first left inferior molar. This tooth was badly broken, the fissure on the inside extending far below the gum.

(TO BE CONTINUED.)

